# Oclusión crónica total coronaria y lesiones en bifurcaciones, lecciones del manejo contemporáneo: reporte de caso

**DOI:** 10.47487/apcyccv.v1i4.87

**Published:** 2020-12-31

**Authors:** Carlos Esteban Uribe-Londoño, Cristhian Felipe Ramirez-Ramos, Gustavo Castilla-Agudelo, Mateo Aranzazu-Uribe, Diego Mauricio Vanegas-Cardona

**Affiliations:** 1 Departamento de Cardiología Intervencionista y Hemodinámica, Cardiovascular Clínica CardioVID. Medellín, Colombia. Departamento de Cardiología Intervencionista y Hemodinámica Cardiovascular Clínica CardioVID Medellín Colombia; 2 Departamento de Cardiología Clínica, Clínica CardioVID y Universidad Pontificia Bolivariana. Medellín, Colombia. Universidad Pontificia Bolivariana Departamento de Cardiología Clínica Clínica CardioVID Universidad Pontificia Bolivariana Medellín Colombia; 3 Departamento de Medicina Interna, Universidad Pontificia Bolivariana. Medellín, Colombia. Universidad Pontificia Bolivariana Departamento de Medicina Interna Universidad Pontificia Bolivariana Medellín Colombia

**Keywords:** Enfermedad Coronaria, Dolor en el Pecho, Angiografía Coronaria, Coronary Disease, Chest Pain, Coronary Angiography

## Abstract

La enfermedad coronaria que involucra las oclusiones crónicas y las lesiones en bifurcación continúa siendo un reto para el cardiólogo intervencionista. La mejora en las técnicas ha permitido tener una mayor tasa de éxito; sin embargo, la mejor estrategia de intervención es desconocida en este subgrupo de pacientes con oclusiones crónicas y lesiones en bifurcaciones asociadas. Presentamos el caso de un paciente en quien, en una angiografía por estudio de dolor torácico, se evidencia una oclusión total crónica y una lesión en bifurcación que fueron tratadas de manera exitosa por intervencionismo coronario.

## Abstract (en)

Coronary artery disease involving chronic occlusions and bifurcation lesions continues to be a challenge for the interventional cardiologist. The improvement in the techniques has allowed a higher success rate, however, the best intervention strategy is unknown in this subgroup of patients with chronic occlusions and associated bifurcation lesions. We present the case of a patient in whom, in an angiography for study of chest pain, a chronic total occlusion and a bifurcation lesion were evidenced and were successfully treated by coronary intervention.

Las oclusiones crónicas totales (OCT) son arterias coronarias completamente obstruidas con flujo TIMI 0 (TIMI, Thrombolysis In Myocardial Infarction) por un tiempo estimado de al menos tres meses^1^. En años recientes, la tasa de éxito de la revascularización de OCT por intervención coronaria percutánea (ICP) ha mejorado sustancialmente, junto con el refinamiento de las indicaciones, la mejoría del material y las técnicas empleadas ^1^. Esto se ha asociado con una mejoría en la calidad de vida, pero con datos contradictorios con relación al pronóstico y la función cardiaca ^(^^2^. Por otro lado, las lesiones en bifurcaciones (LB) suman el 20% de las lesiones tratadas con angioplastia percutánea ^3^, representando un tipo de lesión distinta con mayor riesgo de complicaciones relacionadas con el procedimiento, como los infartos periprocedimiento ^4^. El escenario en el que coexista una OCT y una LB implica un reto importante para el cardiólogo intervencionista, con datos poco claros que guíen el abordaje apropiado, así como los resultados a corto y largo plazo.

Presentamos el caso de un paciente intervenido de manera electiva y de forma exitosa por una OCT en una LB. El objetivo de presentar este caso es destacar la evolución que ha tenido la ICP en el tratamiento de estas lesiones, hecho que ha permitido tratar exitosamente pacientes de complejidad mayor.

## Descripción del caso

Masculino de 76 años, con historia de hipertensión arterial sistémica, prediabetes y extabaquismo, que fue evaluado en la consulta externa por un cuadro de cuatro meses de evolución de disnea de moderados esfuerzos con una clase funcional NYHA II asociado con malestar torácico que mejoraba con el reposo. El paciente presentaba historia de enfermedad coronaria diagnosticada en el contexto de un infarto agudo del miocardio, con elevación del segmento ST inferior, en el año 2002, por compromiso de arteria circunfleja (aCX) con una oclusión en el tercio medio luego de originar la primera obtusa marginal (OM1) que tenía un compromiso proximal del 99%; el manejo del evento agudo consistió en angioplastia percutánea de tercio medio y distal del vaso culpable, sin implante de STENT por motivos no claros y sin intervenir la lesión de la OM1. El paciente permaneció asintomático hasta el cuadro clínico actual. Se le realizó una perfusión miocárdica con dipiridamol que mostró una hipoperfusión severa de la zona inferobasal que revertía en el reposo con una extensión del 12%. El ecocardiograma basal mostró un ventrículo izquierdo de tamaño normal, con contractilidad conservada y una fracción de eyección izquierda en 65%.

**Figura 1:**
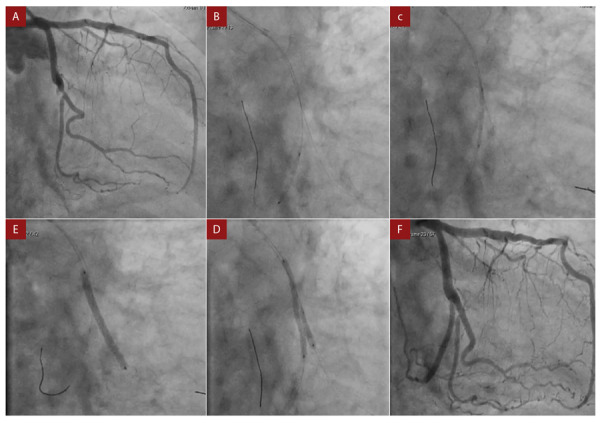
Angiografía coronaria diagnóstica y procedimiento de intervención. A) Angiografía diagnóstica que muestra oclusión total distal de la arteria circunfleja con lesión del 80% de la arteria primara obtusa marginal. B) y C) Angioplastia sobre arteria circunfleja (video 3). D) Angioplastia e implante de stent sobre la arteria primera diagonal E) Kissing balloon sobre arteria circunfleja y primera diagonal. F) Resultado angiográfico final con recanalización sobre arteria circunfleja con mejoría de la lesión de la bifurcación sobre la primera diagonal, flujo TIMI III.

El estudio angiográfico mostró que la aCX tenía una oclusión total crónica en su segmento distal, la OM1 era bifurcada con lesión del 80% (Figura 1a, video1). Las demás arterias no tenían lesiones significativas; durante el procedimiento se procedió de la siguiente manera: utilizando un catéter guía XB3.5 de 6F con microcateter caravel se avanzó guía RUNTHROUGH de 0,014” en OM1 y luego una guía 0,014” Hi-Torque PILOT 200 con técnica de AWE (*antegrade wire escalation*, escalonamiento anterógrado de guías) posicionándola en el tercio distal; luego se realizó con angioplastia con balón MINI TREK complaciente de 2x20 a 10 atm (Figura 1b-c, video 2).

Posterior a esto se realizó una angioplastia con STENT medicado Resolute Onyx (Zotarolimus) 2,5x38 a 18 atm (Figura 1d**, video 3)** desde la OM1 a la aCX y luego se realizó angioplastia con balón complaciente TREK 4x20 a 10 atm con optimización proximal, logrando un flujo TIMI III. Luego, con técnica de TAP se implanta un *stent* medicado Onyx 2,25x34 a 18 atm; finalmente se realizó *kissing balloon* con balones 2,5x15 **(**Figura 1e**, video 4)** logrando un flujo TIMI III **(**Figura 1f**, video 5)**; no se presentaron complicaciones.

Luego de vigilancia durante 24 h en la unidad de cuidado coronario, fue dado de alta para continuar manejo de prevención secundaria. 

## Discusión

En el presente caso se ejemplifica cómo con el tiempo se han incrementado los pacientes con lesiones complejas, en particular, destacado aquí, un caso de coexistencia de OCT y LB. El paciente fue diagnosticado con enfermedad coronaria en un evento con elevación del segmento ST (con una obstrucción aguda de la aCX) hace 18 años, cuando ya presentaba una lesión en la bifurcación de la primera OM; en esa ocasión se manejó con angioplastia del vaso culpable sin implante de *stent*, sin intervenir la OM1 a criterio del grupo intervencionista por el tamaño del vaso. Ahora, en el contexto de una angina estable, se evidencia progresión de la enfermedad coronaria ya con una oclusión crónica de la arteria circunfleja y una LB de la OM1: un verdadero reto desde el punto de vista de manejo percutáneo.

La OCT es el subtipo más complejo de lesión para ICP, con una tasa de éxito baja comparada con las de otro tipo ^1^^,^^5^; su prevalencia oscila entre 18-52% ^6^ dependiendo de la presentación clínica por la que se indique la angiografía. Los datos del estudio de oclusiones crónicas de Canadá con 14 439 pacientes, mostraron una incidencia de 18% en pacientes con enfermedad coronaria conocida ^7^. La prevalencia de OCT en pacientes que son llevados a coronariografía por un infarto agudo del miocardio con elevación del ST es del 21% en diabéticos y 12% en no diabéticos ^8^.

En el estudio HORIZONS-AMI ^9^, la presencia de OCT en una arteria no relacionada con el infarto fue un predictor de muerte a tres años de seguimiento. Otro estudio demostró que la presencia de OCT se asocia con un aumento de la mortalidad durante el seguimiento, al excluir las causas tempranas de muerte ^10^. La revascularización exitosa de las OCT ha demostrado que reduce casi 48% la mortalidad, al compararlo con no tener éxito en la misma ^11^. Sin embargo, pocos estudios han mostrado mejoría en la mortalidad en los pacientes llevados a ICP por OCT, comparado con la terapia médica, pero sí se ha demostrado un beneficio en la calidad de vida^12^. Se destaca el hecho que la tasa de eventos cardiovasculares en las pruebas ha sido bajo (6,7% en el grupo terapia médica vs. 5,2% en grupo ICP) lo que tal vez explique la falta de beneficio en mortalidad. Hay siete principios básicos que tener presentes cuando se realiza la PCI de OCT (Tabla 1) ^(^^1^.

**Tabla 1:** Principios claves en las indicaciones y técnica de OCT

1	La principal indicación de ICP de una OCT es la mejoría de los síntomas.
2	La angiografía coronaria dual y una vista estructurada y detallada debe realizarse en cada caso.
3	El uso de microcatéteres es esencial para el soporte de guías.
4	Las cuatro estrategias de cruce son: escalonamiento anterógrado de guías (AWE), reentrada/disección anterógrada, escalonamiento retrogrado de guías (RWE) y la disección/reentrada retrógrada.
5	El cambio del equipo y la técnica incrementa la probabilidad de éxito y mejora la eficiencia del procedimiento
6	Los centros y los médicos que realizan ICP de OCT deben tener el equipo, la experiencia y la experticia para optimizar los resultados y minimizar como manejar las complicaciones.
7	Se debe hacer los esfuerzos necesarios para implantar de manera adecuada los stents en la ICP de una OCT, incluyendo la utilización de imágenes intravasculares.

Adaptado de. Brilakis et al. Circulation. 2019;140:420-433- ICP: intervención coronaria percutánea. OCT: oclusiones crónicas totales.

Por otro lado, las LB son reportadas en 15-20% de las lesiones tratadas de manera percutánea ^3^. En general para las lesiones limitadas a los 5 mm del ostium, se puede emplear una técnica de *stent* provisional. Después de predilatar el vaso principal, se debe emplear un *stent* largo que permita la optimización proximal, con un diámetro basado en el lumen distal lo que admite que no se sobredimensione el *stent* en la carina de la lesión y así reducir el riesgo de desplazamiento de la placa. La técnica de optimización proximal (POT, *Proximal Optimization Technique*) mejora la expansión del *stent* y facilita el recruce de la rama lateral, si es necesario. La ventaja fundamental de la aproximación provisional es que el tratamiento de la rama lateral permanece como una opción en cualquier momento del procedimiento ^(^^13^. En las ramas laterales pequeñas con flujo reducido, el recruce con la guía por el *strut*, con posterior dilatación de la rama lateral y optimización proximal, debe considerarse de manera inicial ^(^^13^. El implante de un segundo *stent* se basa en el tamaño del vaso y en los resultados angiográficos logrados. El uso de un segundo *stent* puede ser necesario en el 10% de los pacientes tratados con la técnica previsional ^(^^13^. Si persiste el compromiso de la rama lateral con flujo disminuido, se puede utilizar una estrategia de 2-*stent* en T, TAP (*stent* en T y protrusión) o cullote ^6^. La elección de la técnica de *stents* en T o TAP, puede basarse en imágenes que permitan una adecuada visualización del *stent* para valorar la presencia de malposición en la rama principal opuesta a la rama lateral en la pared opuesta de la carina después del *Kissing* y la optimización proximal ^13^. En estas circunstancias la técnica de *stent* en T debe ser considerada. Por otro lado, el tratamiento en lesiones con anatomía compleja y compromiso ateroesclerótico difuso, tanto de la rama principal como de la lateral, es más probable que requiera una aproximación de 2 *stent*^13^. El uso electivo de la técnica de 2 *stent* está indicada en lesiones muy complejas con calcificación de la rama lateral o una enfermedad ostial que va >5 mm de la carina, además en lesiones de bifurcaciones con una rama lateral mayor cuyo acceso sea particularmente un reto ^13^. Las técnicas de 2 *stent* no se utilizan como estrategia inicial con relación a la provisional, por el mayor riesgo de trombosis del *stent*^6^^,^^13^^,^^14^. 

La incidencia reportada de LB en contexto de OCT oscila entre el 26-47%; sin embargo, se desconoce cuál es la mejor aproximación de tratamiento ^14^. En este caso se utilizó una aproximación de intervención de implante de *stent* en la lesión de bifurcación dirigido hacia el vaso principal con técnica de optimización, para luego proceder con la intervención de la OCT, con optimización proximal y con *Kissing* balón en los dos vasos, con un buen resultado angiográfico.

La disección durante la recanalización podría requerir de *stent* en la rama lateral, algo desaconsejado en las lesiones no-OCT. La predilatación de la rama lateral antes del *stent* en el vaso principal se ha asociado con un incremento de flujo, pero con un mayor riesgo de disección ostial del vaso lateral; sin embargo, en las OCT esta preocupación es menos importante, pues la disección del vaso lateral frecuentemente ocurre antes de la bifurcación.

De manera interesante Ojeda *et al.* encontraron una incidencia de 33% de lesiones bifurcadas con OCT, con una tasa de 94% de utilización de *stent* provisional, con un éxito del 81% de las lesiones en bifurcación; los predictores de éxito fueron la guía de la rama lateral y la ausencia de disección a través de la bifurcación. Los pacientes con flujo TIMI <III tuvieron mayor incidencia de infarto periprocedimental (32% vs. 4,8%), pero en el seguimiento no se encontraron diferencias en esta tasa de eventos (7,7% vs. 9,5%); sus resultados sugieren que una aproximación regular para las lesiones de bifurcaciones puede ser utilizada en el contexto de OCT; sin embargo, en nuestro caso por el tamaño del vaso se decidió implantar *stent* para protegerlo y luego poder intervenir la obstrucción crónica.

## Conclusiones

El aumento en la complejidad de las lesiones de enfermedad coronaria, más cuando coexisten, representan un verdadero reto en la intervención coronaria percutánea. La mejoría en los materiales, así como el refinamiento de las técnicas, han permitido lograr tratamientos exitosos. El mejor abordaje de las lesiones en bifurcaciones en pacientes con obstrucciones totales crónicas es desconocido. El uso de *stent* provisional en las lesiones bifurcadas, cuando se realiza el manejo de las oclusiones crónicas totales, podría ser una estrategia de manejo apropiada. 
